# Supplementary material for: Identification of Freezing-Responsive microRNAs and Their Targets in Chinese Jujube by Small RNA and Degradome Sequencing
Source: Int J Mol Sci. 2026 Sep 4;27(17):7912. doi: 10.3390/ijms27177912 (PMC13566890; doi:10.3390/ijms27177912)
Supplement: Supplementary file 1 [file ijms-27-07912-s001.zip › Supplementary Material S2/novel_miR_48.pdf]

Provisional ID	:	novel_miR_48
Score total	:	0.4
Score for star read(s)	:	-1.3
Score for read counts	:	0
Score for mfe	:	0.1
Score for randfold	:	1.6
Score for cons. seed	:	
Total read count	:	61
Mature read count	:	61
Loop read count	:	0
Star read count	:	0

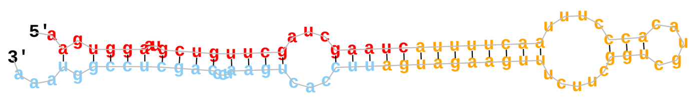

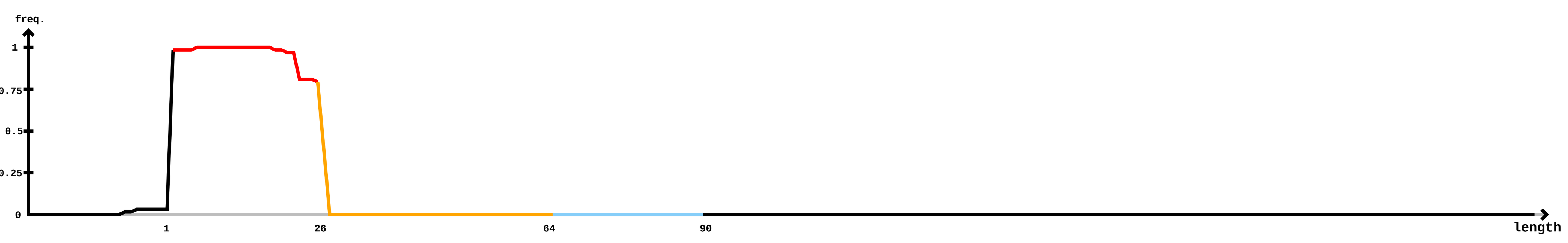


```
                      Mature                                            Star
5'- aaaugguaaaacaugaugcuuuaaguggaaugcuguucgaucgaaucauuuucaauuucccacaugcuggcuucuuugaagaugauuccacugaaucccagcuccgguaaauguuaauauuuuuacaaauuauuuaauaauucuacuaucauuuuaauuucuuaaaauuuaaugaguguuauaaucaaaauuucuauguuuugauaauuauaaucuacgugcaguagaaaccaaaugcuaguagcguugu   -3'   exp
    .....((((((......((.((.(.((((..((((((((...(((((((((((((....(((.....))).....)))))))))))))...))))...)))))))).).)).)).......))))))..........(((((.((((((.(((((......((((........))))..(((((((((((((......)))))))))...)))))(((((.....)))))....))))).))))))).)))))   reads   mm   sample
    .............gaugcuuuaaguggaaugcuguucgau..................   1   0   LT-3
    ...............ugcuuuaaguggaaugcuguucg....................   1   0   LT-3
    ......................aaguggaaugcuguucgaucg...............   10   0   LCK-3
    ......................aaguggaaugcuguucgaucgaauc...........   3   0   LT-2
    ......................aaguggaaugcuguucgaucgaauc...........   22   0   LCK-1
    ......................aaguggaaugcuguucgaucgaauc...........   23   0   LCK-2
    ......................aaguggaaugcuguNcgaucgaauc...........   1   1   LCK-1
    ......................aaguggaCugcuguucgaucgaauc...........   1   1   LCK-1
    ..........................Agaaugcuguucgaucgaau............   1   1   LT-1
```
